# Risk-adapted treatment reduced chemotherapy exposure for clinical stage I pediatric testicular cancer

**DOI:** 10.1186/s12911-020-01365-x

**Published:** 2020-12-14

**Authors:** Yun-lin Ye, Zhuang-fei Chen, Jun Bian, Hai-tao Liang, Zi-ke Qin

**Affiliations:** 1 Department of Urology, Sun Yat-Sen University Cancer Center, State Key Laboratory of Oncology in South China, Collaborative Innovation Center for Cancer Medicine, Guangzhou, 510060 Guangdong China; 2 Department of Urology, Nanfang Hospital, Southern Medical University, Guangzhou, 510515 China; 3 Department of Urology, The Fifth Affiliated Hospital of Guangzhou Medical University, Guangzhou, 510090 Guangdong China

## Abstract

**Background:**

Different from adult clinical stage I (CS1) testicular cancer, surveillance has been recommended for CS1 pediatric testicular cancer. However, among high-risk children, more than 50% suffer a relapse and progression during surveillance, and adjuvant chemotherapy needs to be administered. Risk-adapted treatment might reduce chemotherapy exposure among these children.

**Methods:**

A decision model was designed and calculated using TreeAge Pro 2011 software. Clinical utilities such as the relapse rates of different groups during surveillance or after chemotherapy were collected from the literature. A survey of urologists was conducted to evaluate the toxicity of first-line and second-line chemotherapy. Using the decision analysis model, chemotherapy exposure of the risk-adapted treatment and surveillance strategies were compared based on this series of clinical utilities. One-way and two-way tests were applied to check the feasibility.

**Results:**

In the base case decision analysis of CS1 pediatric testicular cancer, risk-adapted treatment resulted in a lower exposure to chemotherapy than surveillance (average: 0.7965 cycles verse 1.3419 cycles). The sensitivity analysis demonstrated that when the relapse rate after primary chemotherapy was ≤ 0.10 and the relapse rate of the high-risk group was ≥ 0.40, risk-adapted treatment would result in a lower exposure to chemotherapy, without any association with the proportion of low-risk patients, the relapse rate of the low-risk group, the relapse rate after salvage chemotherapy or the toxicity utility of second-line chemotherapy compared to first-line chemotherapy.

**Conclusions:**

Based on the decision analysis, risk-adapted treatment might decrease chemotherapy exposure for these high-risk patients, and an evaluation after orchiectomy was critical to this process. Additional clinical studies are needed to validate this statement.

## Background

Despite the low incidence of pediatric testicular tumors, yolk sac tumors are the most common malignant type in children, which are very different from their adult counterparts [1–6]. Approximately 70% to 80% of pediatric patients have clinical stage I (CS1) disease, and due to its hematogenous predilection for metastasis in children, primary retroperitoneal lymph node dissection (RPLND) is not recommended for CS1 yolk sac tumors [1, 6, 7]. In a recent summary of the PDQ Pediatric Treatment Editorial Board and based on the recommendations of the POG/CCG, surveillance is recommended for children with CS1 testicular cancer after radical inguinal orchiectomy (RIO) [8, 9].

In recent studies, approximately 20% of children with CS1 testicular germ cell tumors (GCT) have suffered a relapse within 4 years after RIO, and they underwent 3–4 cycles of salvage chemotherapy [1, 9]. Advanced analysis demonstrated that an age > 10 years, mixed histology and lymphovascular invasion (LVI) were associated with disease relapse [10, 11]. In high-risk children, more than 50% of them suffered a relapse and progression [6, 10]. Among their adult counterparts, risk-adapted management has achieved a favorable outcome for CS1 testicular nonseminomatous germ cell tumors (NSGCT) [12, 13]. This procedure might also be feasible for pediatric patients and reduce their exposure to chemotherapy, and their outcomes are excellent with surveillance and salvage chemotherapy. However, no study has directly compared the cost and toxicity between surveillance and risk-adapted management.

In this study, using a decision analysis model, we evaluated the chemotherapy burden of CS1 pediatric testicular cancer between risk-adapted treatment and surveillance.

## Methods

**Fig. 1:**
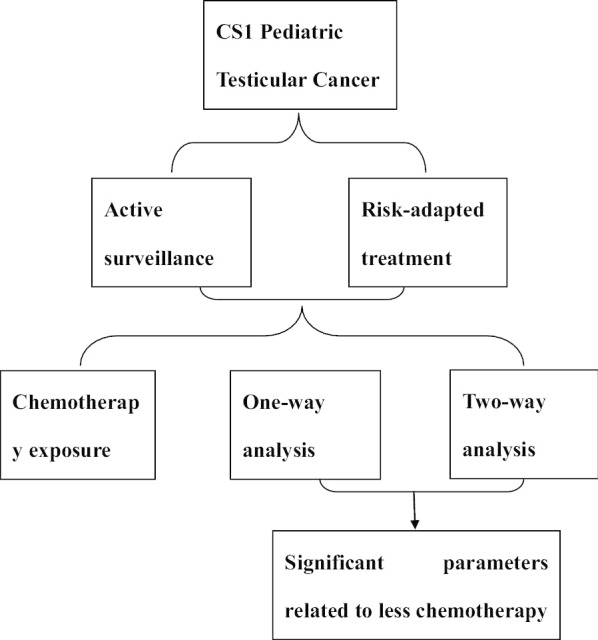
Flowchart of this decision analysis

**Fig. 2:**
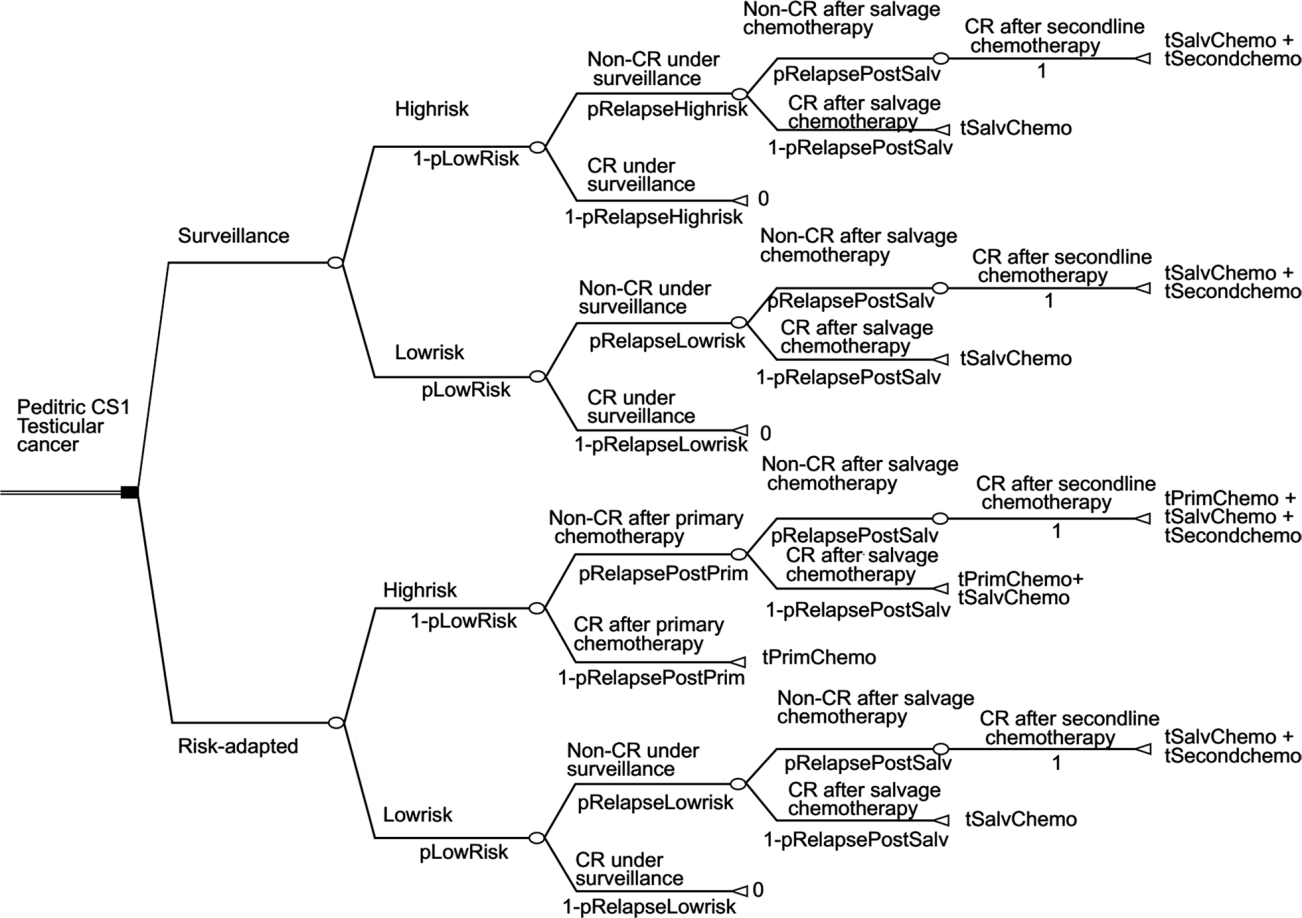
Decision analysis tree of risk-adapted treatment and surveillance

The decision model was designed and calculated using TreeAge Pro 2011 Software (http://www.treeage.com), and the decision trees of the surveillance and risk-adapted treatment and flowchart of the analysis are listed in Figs. 1 and 2.

For these two groups, the cost of radical inguinal orchiectomy and regular follow-up was similar. In China, the cost of the operation, drugs, enrollment and so forth were generally consistent with the legal regulations in the last decade. Generally, chemotherapy toxicity was associated with the number of chemotherapy cycles. Therefore, we just compared the exposure to chemotherapy between the two groups.

Our analysis consisted of the following hypothetical clinical scenarios for the two groups: First, patients in both groups with CS1 testicular cancer were diagnosed with histopathology, serum markers and imaging. Then, for the surveillance group, patients who suffered a relapse during follow-up received salvage chemotherapy consisting of 3 cycles of PEB (cisplatin, VP-16 and bleomycin) chemotherapy. If a complete response (CR) was not achieved after 3 cycles of PEB, second-line chemotherapy with 3 cycles of VIP (VP-16, ifosfamide and cisplatin) was performed. For the risk-adapted group, the high-risk group received primary chemotherapy with 1 cycle of PEB, and the low-risk group underwent surveillance. Then, salvage chemotherapy with 3 cycles of PEB was performed when a relapse was detected. If a CR was not achieved after 3 cycles of PEB, second-line chemotherapy with VIP was performed.

According to recent studies, the overall survival of CS1 pediatric testicular cancer was nearly 100% with systemic chemotherapy, and the progression rates after primary and salvage chemotherapy were both approximately 5% (2.3–6.8%) (Table 1) [6, 9, 12, 14–20]. The rare cases of an operation or radiation after chemotherapy were reported as recommended by the guidelines.

**Table 1:** Proportions used in decision model

	Point estimate	Range	References
Relapse of low risk group	0.15	0.10–0.20	[6, 9, 12, 14, 15]
Relapse of high risk group	0.60	0.38–0.73	[6, 9, 12, 14]
Progression after primary chemotherapy	0.05	0.01–0.10	[12, 15–18]
Progression after salvage chemotherapy	0.05	0.01–0.22	[6, 9, 14, 16, 18–20]
Progression after second-line chemotherapy	0		
Toxicity of Primary chemotherapy	1		
Toxicity of Salvage chemotherapy	3 × 1		
Toxicity of Second-line chemotherapy	3 × 1.3	3 × 1.0–3 × 2.0	Interview

Based on these studies, we defined the relapse rates of high- and low-risk patients who underwent surveillance as 0.60 (0.38–0.73) and 0.15 (0.10–0.20), respectively; the proportion of low-risk patients was 0–1, the progression rates after primary and salvage chemotherapy were both 0.05 (0.01–0.10), and second-line chemotherapy was the last treatment with a 100% success rate (as shown in Fig. 2 and Table 1) [6, 9, 12, 14–20].

To evaluate treatment-related toxicity between first-line and second-line chemotherapy, digital values were obtained in an interview with urological oncologists. Before the interview, the consensus about short- and long-term toxicity of chemotherapy for testicular cancer was acquired. By means of a visual analog scale, compared to surveillance, values of salvage chemotherapy and second-line chemotherapy were assessed as 0.841 (95% confidence interval: 0.811–0.871) and 0.635 (95% confidence interval: 0.578–0.697), respectively. Therefore, we defined the toxicity of second-line chemotherapy as approximately 0.814/0.635 = 1.3 times that of salvage chemotherapy. The range was defined as 1.0–2.0 in the decision analysis.

## Results

In all, 24 urologists and oncologists took part in the interview to evaluate the toxicity of chemotherapy for pediatric testicular cancer. As shown in Table 2, compared to orchiectomy without chemotherapy (value = 1.0), the value of first-line chemotherapy was from 0.682–1.000, and the value of second-line chemotherapy was from 0.435–0.960. The average number and standard deviation were 0.841 and 0.081, and 0.635 and 0.151, respectively. We defined the toxicity of second-line chemotherapy as approximately 0.814/0.635 = 1.3 times that of salvage chemotherapy, ranging from 1.0 to 2.0 in the decision analysis.

**Table 2:** Results of survey for chemotherapy toxicity

No	No chemotherapy	First-line chemotherapy		Second-line chemotherapy	
			Relative value		Relative value
1	95	85	0.895	75	0.789
2	95	75	0.789	50	0.526
3	80	70	0.875	65	0.813
4	95	70	0.737	50	0.526
5	95	70	0.737	50	0.526
6	90	70	0.778	50	0.556
7	95	80	0.842	50	0.526
8	100	80	0.8	60	0.6
9	90	85	0.944	60	0.667
10	95	80	0.842	65	0.684
11	90	80	0.889	60	0.667
12	90	70	0.778	50	0.556
13	90	80	0.889	70	0.778
14	85	58	0.682	37	0.435
15	100	90	0.9	87	0.87
16	100	100	1	96	0.96
17	100	99	0.99	93	0.93
18	95	70	0.737	45	0.474
19	100	80	0.8	50	0.5
20	85	70	0.824	50	0.588
21	95	75	0.789	50	0.526
22	90	80	0.889	40	0.444
23	95	85	0.895	60	0.632
24	90	80	0.889	60	0.667
Average			0.841		0.635
SD			0.081		0.151

**Fig. 3:**
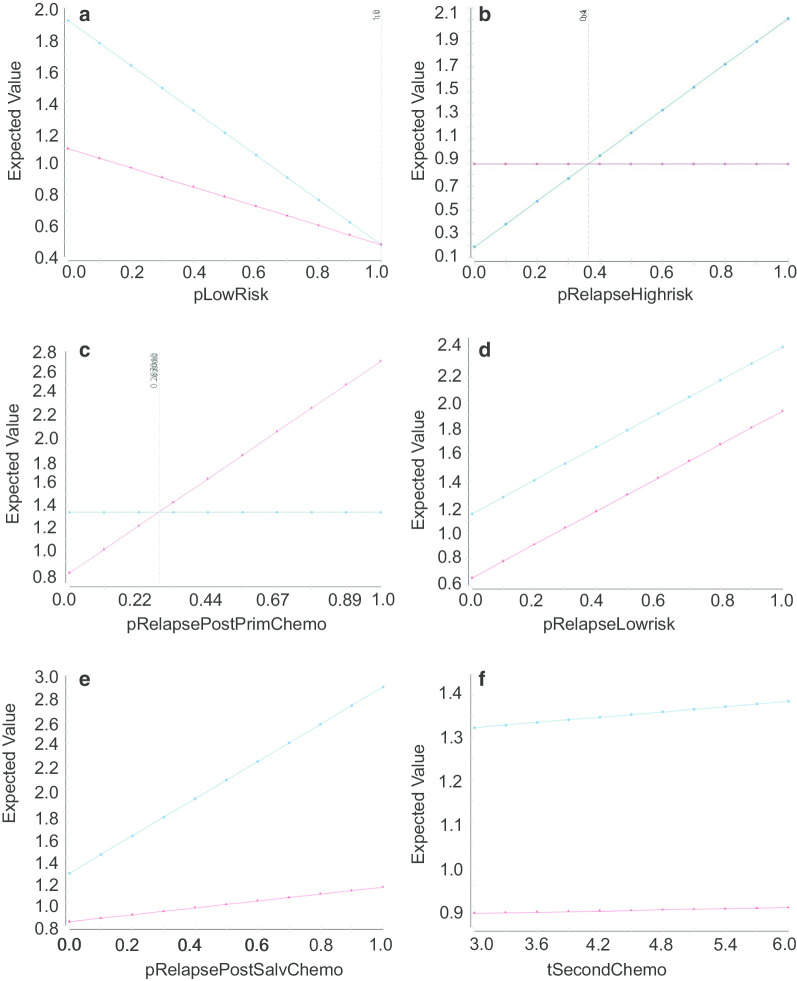
1-way sensitivity analysis. **a** In any value of pLowRisk (proportion of low-risk patients), surveillance was associated with higher exposure of chemotherapy; **b** When pRelapseHighrisk (relapse rate of high-risk group) > 0.365, surveillance was associated with higher exposure of chemotherapy; **c** When pRelapsePostPrimChemo (relapse rate after primary chemotherapy) < 0.287, surveillance was associated with higher exposure of chemotherapy; **d** in any value of pRelapseLowrisk (relapse rate of low-risk group), surveillance was associated with higher exposure of chemotherapy; **e** in any value of pRelapsePostSalvChemo (relapse rate after salvage chemotherapy), surveillance was associated with higher exposure of chemotherapy; **f** in any value of tSecondChemo (toxicity utility of second-line chemotherapy compared to salvage chemotherapy), surveillance was associated with higher exposure of chemotherapy. Red: risk-adapted treatment, blue: surveillance

Our analysis demonstrated that risk-adapted treatment resulted in a lower exposure to chemotherapy than surveillance (average: 0.7965 cycles verse 1.3419 cycles). A 1-way sensitivity analysis demonstrated that the differences in chemotherapy exposure between the two treatments were associated with the proportion of low-risk patients (pLowRisk): when pLowRisk = 0, all of the patients were in the high-risk group, and the two treatments had significantly different exposures to chemotherapy; when pLowRisk = 1, all patients were in the low-risk group, and the two groups had the same exposure to chemotherapy (Fig. 3a). Similarly, when the relapse rate of the high-risk group (pRelapseHighrisk) was ≥ 0.40 and relapse rate after primary chemotherapy (pRelapsePostPrimChemo) was ≤ 0.25, risk-adapted treatment was associated with lower chemotherapy exposure (Fig. 3b, c). Risk-adapted treatment was associated with lower chemotherapy exposure without association with the relapse rate of the low-risk group (pRelapseLowrisk), the relapse rate after salvage chemotherapy (pRelapsePostSalvChemo) and the toxicity utility of second-line chemotherapy compared to salvage chemotherapy (tSecondChemo) (Fig. 3d–f). This means only pRelapseHighrisk and pRelapsePostPrimChemo were associated with the utility of chemotherapy exposure, so we focused on these two factors in the 2-way sensitivity analysis.

**Fig. 4:**
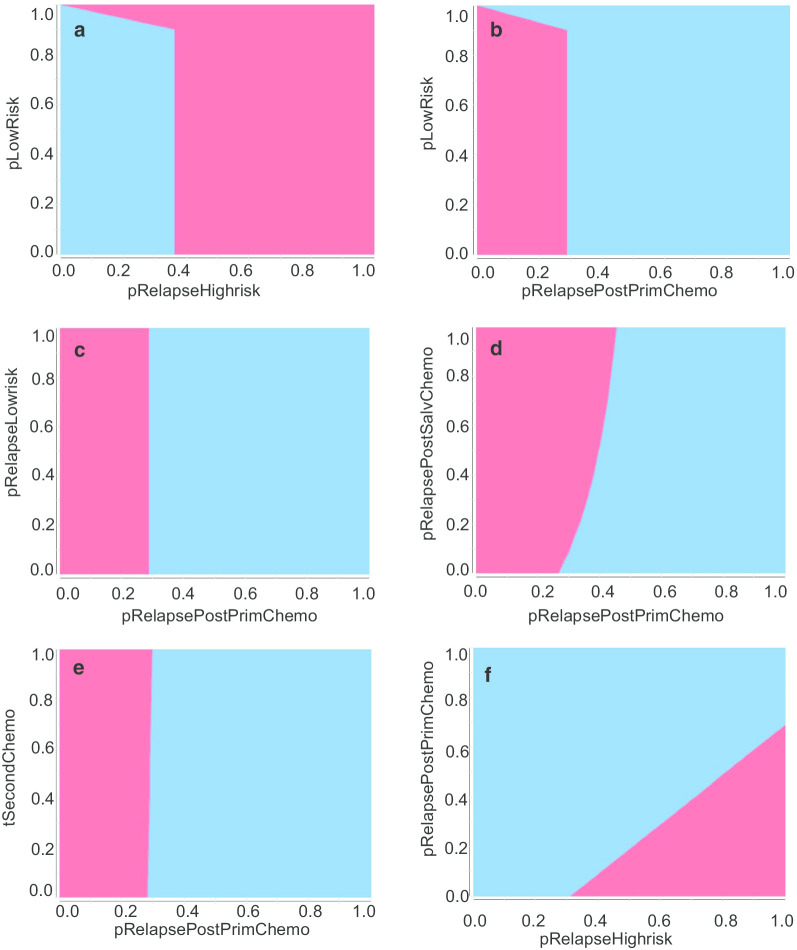
2-way sensitivity analysis. **a** In any value of pLowRisk (proportion of low-risk patients), when pRelapseHighrisk (relapse rate of high-risk group) > 0.365, risk-adapted treatment was associated with lower exposure of chemotherapy; **b** in any value of pLowRisk (proportion of low-risk patients), when pRelapsePostPrimChemo (relapse rate after primary chemotherapy) < 0.287, risk-adapted treatment was associated with lower exposure of chemotherapy; **c** in any value of pRelapseLowrisk (relapse rate of low-risk group), when pRelapsePostPrimChemo (relapse rate after primary chemotherapy) < 0.287, risk-adapted treatment was associated with lower exposure of chemotherapy; **d** in any value of pRelapsePostSalvChemo (relapse rate after salvage chemotherapy), when pRelapsePostPrimChemo (relapse rate after primary chemotherapy) < 0.287, risk-adapted treatment was associated with lower exposure of chemotherapy; **e** in any value of tSecondChemo (toxicity utility of second-line chemotherapy compared to salvage chemotherapy), when pRelapsePostPrimChemo (relapse rate after primary chemotherapy) < 0.287, risk-adapted treatment was associated with lower exposure of chemotherapy; **f** when pRelapsePostPrimChemo (relapse rate after primary chemotherapy) < 0.1, and pRelapseHighrisk (relapse rate of high-risk group) > 0.4, risk-adapted treatment was associated with lower exposure of chemotherapy. Red: risk-adapted treatment, blue: surveillance

In the 2-way sensitivity analysis, we found that when the pRelapseHighrisk was ≥ 0.40, risk-adapted treatment was associated with lower chemotherapy exposure without an association with pLowRisk, pRelapseLowrisk, pRelapsePostSalvChemo or tSecondChemo (Fig. 4a, Additional file 1: Fig. 1A, B, C). When pRelapsePostPrimChemo was ≤ 0.25, and pLowRisk was ≤ 0.90, risk-adapted treatment was associated with less chemotherapy exposure (Fig. 4b). When pRelapsePostPrimChemo was ≤ 0.25, risk-adapted treatment would result in a lower exposure to chemotherapy without an association with pRelapseLowrisk, pRelapsePostSalvChemo or tSecondChemo (Fig. 4c–e). In the 2-way sensitivity analysis of pRelapseHighrisk and pRelapsePostPrimChemo, when pRelapsePostPrimChemo was ≤ 0.10 and pRelapseHighrisk was ≥ 0.40, risk-adapted treatment would result in a lower exposure to chemotherapy (Fig. 4f).

## Discussion

Since pediatric testicular cancer is generally universally curable, surveillance is recommended for clinical stage 1 patients, and salvage chemotherapy is given when relapsed disease is detected [8, 9]. In their adult counterparts, risk-adapted management has favorable outcomes, and decision analysis has demonstrated that surveillance is the preferred intervention, except for those patients with a high risk of relapse [12, 13]. Meanwhile, due to the extremely long survival time of these pediatric patients, treatment-related toxicity also should be taken into consideration [21]. In some studies, primary chemotherapy was associated with an extremely low relapse rate, and it decreased the relapse rate in the high-risk group significantly [6]. Therefore, we used decision analysis to develop a model to evaluate the chemotherapy exposure between the two protocols. Risk-adapted management might reduce the exposure to chemotherapy by primary chemotherapy among high-risk patients.

TreeAge Pro is the leading software for decision analysis, and the decision model was developed based on historical data from the previous literature. Although this model is simple, the exposure to chemotherapy could be clearly calculated. Several prediction methods based on artificial intelligence have been developed, but clouds of data or lots of instruments are needed [22–24]. For this rare cancer, which is sporadic, big data are not available. Therefore, we chose a simple decision model focused on chemotherapy exposure.

In this study, risk-adapted treatment resulted in less exposure to chemotherapy than surveillance, which is not consistent with the clinical decisions made by following the current guidelines. In the 1-way sensitivity analysis, only the relapse rate of the high-risk group (pRelapseHighrisk) and the relapse rate after primary chemotherapy (pRelapsePostPrimChemo) were associated with chemotherapy exposure. When pRelapseHighrisk was ≥ 0.40 or pRelapsePostPrimChemo was ≤ 0.25, risk-adapted treatment resulted in lower chemotherapy exposure, and these two utilities are reasonable in clinical practice (Fig. 3). Within a 2-way analysis, when pRelapsePostPrimChemo was ≤ 0.10 and pRelapseHighrisk was ≥ 0.40, risk-adapted treatment would decrease chemotherapy exposure, without any association with the other four factors. These results implied that with the more precise stratification of the high-risk group and the higher CR rate of primary chemotherapy, better individualized management would be accomplished, and less treatment-related toxicity would occur.

In recent studies, the rate of relapse was approximately 20% for CS1 pediatric testicular cancer, and most cases occurred in the first 2 years [9]. In some limited series, the relapse rate of the high-risk group was approximately 60%, and that of the low-risk group was 15% [6, 9, 12, 14–20]. The relapse rate of patients with primary chemotherapy was less than 5% and the overall survival rate was nearly 100%. In our prior study, the relapse rate was approximately 33% and the overall survival was 98%. Meanwhile, necrosis, a new predictor of tumor relapse, when combined with LVI stratified the patients into 2 groups, and the relapse rates were 73% and 17%, respectively [6]. In other studies, the relapse rate of the high-risk group was 0.38–0.55, and the relapse rate of the low-risk group was 0.16–0.19 [9, 12, 14–20]. Based on these data, we found that the chemotherapy exposure was lower in the risk-adapted treatment in our model. Due to the favorable outcome of salvage chemotherapy for clinical stage 1 patients, primary chemotherapy was not common in these studies. However, some studies also demonstrated that primary chemotherapy was associated with an extremely low relapse rate [6]. In adult patients with CS1 testicular NSGCT, primary chemotherapy achieved an excellent oncological outcome and this procedure might also be effective in pediatric patients [12, 13].

Actually, based on the contemporary scenario, this study revealed that risk-adapted treatment was associated with significantly less chemotherapy exposure. pRelapsePostPrimChemo and pRelapseHighrisk were significant factors that decreased exposure to chemotherapy, which implied that the effectiveness of primary chemotherapy and the identification of high-risk patients were critical to individualized management. For primary chemotherapy, the outcome is favorable and a lower-toxicity regimen might be available [19]. In a recent study, the relapse rate of the high-risk group was > 70% with a combination of two high-risk factors (LVI and necrosis), and further research into prognostic markers is necessary [6]. As precise management of cancers has developed, the differentiation of boys with testicular cancer into risk groups would allow for more precisely tailored treatment, and risk-adapted treatment would reduce chemotherapy exposure substantially [25].

Our study had some limitations worth noting. To simplify the analysis of chemotherapy toxicity, we calculated cycles of chemotherapy instead of the detailed side effects, such as cardiovascular disease, neurotoxicity, ototoxicity, chronic kidney disease, and infertility. The proportions were defined according to recent studies, but since these cases are rare, bias was present, and some of them were included in studies about their adult counterparts. Due to the shortage of life-long follow-up of this curable disease, quality-adjusted life-years and cost-effectiveness analyses were not performed in this study. Despite these limitations, we believe this model could imply some advantages of risk-adapted management in CS1 pediatric testicular cancer. This is the first report regarding the chemotherapy burden of CS1 pediatric testicular cancer.

## Conclusions

Our decision model of management for clinical stage 1 pediatric testicular cancer demonstrated that risk-adapted treatment was associated with a lower exposure to chemotherapy. Additional clinical studies are needed to validate this statement.

## Supplementary Information

**Additional file 1: Figure 1**. Way sensitivity analysis. A: In any value of pRelapseLowrisk (relapse rate of low-risk group), when pRelapseHighrisk (relapse rate of high-risk group) > 0.4, risk-adapted treatment was associated with lower exposure of chemotherapy; B: In any value of pRelapsePostSalvChemo (relapse rate after salvage chemotherapy), when pRelapseHighrisk (relapse rate of high-risk group) > 0.4, risk-adapted treatment was associated with lower exposure of chemotherapy; C: In any value of tSecondChemo (toxicity utility of second-line chemotherapy compared to salvage chemotherapy), when pRelapseHighrisk (relapse rate of high-risk group) > 0.4, risk-adapted treatment was associated with lower exposure of chemotherapy. Red: risk-adapted treatment, Blue: surveillance.

## Data Availability

Most data was derived from literature as referred in manuscript, and the survey data was shown in Table 2.

## Abbreviations

CS1: Clinical stage 1
RPLND: Retroperitoneal lymph node dissection
POG/CCG: Pediatric Oncology Group and Children's Cancer Group
RIO: Radical inguinal orchiectomy
GCT: Germ cell tumors
LVI: Lymphovascular invasion
NSGCT: Nonseminomatous germ cell tumors
PEB: Cisplatin, VP-16 and bleomycin
CR: Complete response
VIP: VP-16, ifosfamide and cisplatin
pLowRisk: Proportion of low-risk patients
pRelapseHighrisk: Relapse rate of high-risk group
pRelapsePostPrimChemo: Relapse rate after primary chemotherapy
pRelapseLowrisk: Relapse rate of low-risk group
pRelapsePostSalvChemo: Relapse rate after salvage chemotherapy
tSecondChemo: Toxicity utility of second-line chemotherapy compared to salvage chemotherapy
