# Supplementary figures and images for: Risk-adapted treatment reduced chemotherapy exposure for clinical stage I pediatric testicular cancer
Source: BMC Med Inform Decis Mak. 2020 Dec 14;20:337. doi: 10.1186/s12911-020-01365-x (PMC7737364; doi:10.1186/s12911-020-01365-x)

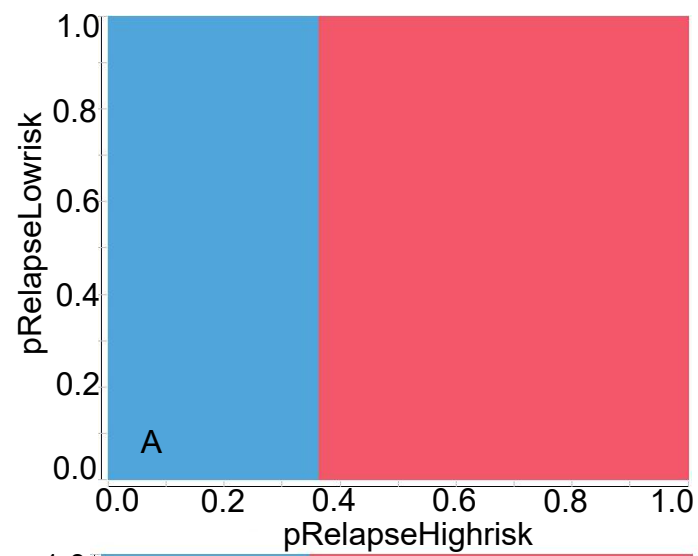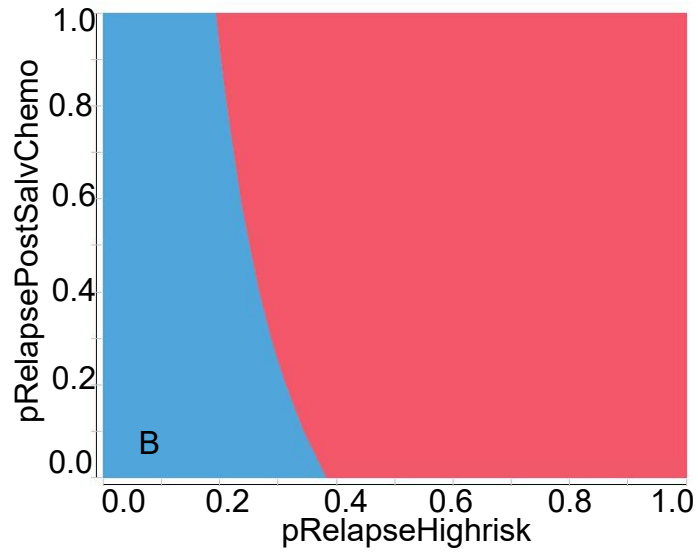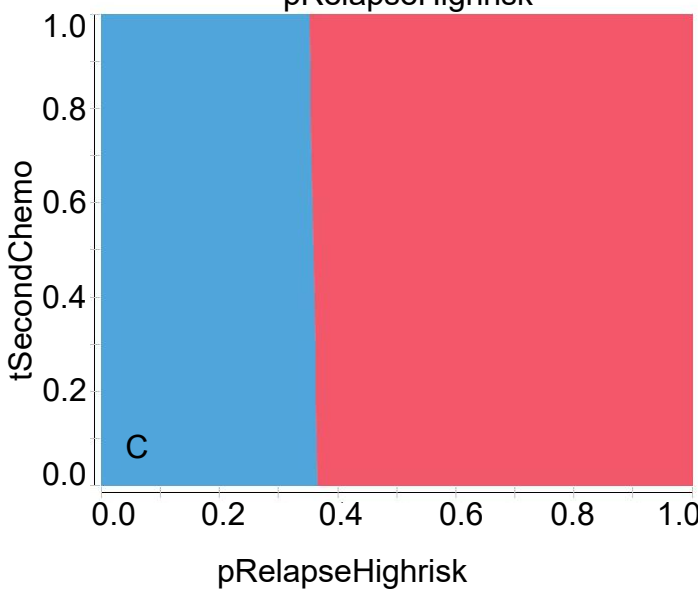

Supplement: Supplementary file 1 — Additional file 1: Figure 1. Way sensitivity analysis. A: In any value of pRelapseLowrisk (relapse rate of low-risk group), when pRelapseHighrisk (relapse rate of high-risk group) > 0.4, risk-adapted treatment was associated with lower exposure of chemotherapy; B: In any value of pRelapsePostSalvChemo (relapse rate after salvage chemotherapy), when pRelapseHighrisk (relapse rate of high-risk group) > 0.4, risk-adapted treatment was associated with lower exposure of chemotherapy; C: In any value of tSecondChemo (toxicity utility of second-line chemotherapy compared to salvage chemotherapy), when pRelapseHighrisk (relapse rate of high-risk group) > 0.4, risk-adapted treatment was associated with lower exposure of chemotherapy. Red: risk-adapted treatment, Blue: surveillance. [file 12911_2020_1365_MOESM1_ESM.pdf]
